# Vaccine Potency and Structure of Yeast-Produced Polio Type 2 Stabilized Virus-like Particles

**DOI:** 10.3390/vaccines12091077

**Published:** 2024-09-20

**Authors:** Qin Hong, Shuxia Wang, Xiaoli Wang, Wenyu Han, Tian Chen, Yan Liu, Fei Cheng, Song Qin, Shengtao Zhao, Qingwei Liu, Yao Cong, Zhong Huang

**Affiliations:** 1 Key Laboratory of RNA Innovation, Science and Engineering, Shanghai Institute of Biochemistry and Cell Biology, Center for Excellence in Molecular Cell Science, Chinese Academy of Sciences, University of Chinese Academy of Sciences, Shanghai 200031, China; hongqin2018@sibcb.ac.cn; 2 Shanghai Institute of Infectious Disease and Biosecurity, Key Laboratory of Medical Molecular Virology (MOE/NHC/CAMS), Shanghai Medical College, Fudan University, Shanghai 200032, China; shuxia_wang@fudan.edu.cn (S.W.); chentian28993@163.com (T.C.); 3 Huasong (Shanghai) Biomedical Technology Co., Ltd., Shanghai 201210, China; xiaoliwang@huasongbio.com (X.W.); yanfa3@huasongbio.com (W.H.); liuyan0848@163.com (Y.L.); qinsong@huasongbio.com (S.Q.); zhaoshengtao@huasongbio.com (S.Z.); 4 Key Laboratory of Systems Health Science of Zhejiang Province, School of Life Science, Hangzhou Institute for Advanced Study, University of Chinese Academy of Sciences, Hangzhou 310024, China

**Keywords:** poliovirus, virus-like particle, immunogenicity, neutralizing antibody, cryo-EM structure

## Abstract

Poliovirus (PV) is on the brink of eradication due to global vaccination programs utilizing live-attenuated oral and inactivated polio vaccines. Recombinant PV virus-like particles (VLPs) are emerging as a safe next-generation vaccine candidate for the impending polio-free era. In this study, we investigate the production, antigenicity, thermostability, immunogenicity, and structures of VLPs derived from PV serotype 2 (PV2) wildtype strain and thermally stabilized mutant (wtVLP and sVLP, respectively). Both PV2 wtVLP and sVLP are efficiently produced in *Pichia pastoris* yeast. The PV2 sVLP displays higher levels of D-antigen and significantly enhanced thermostability than the wtVLP. Unlike the wtVLP, the sVLP elicits neutralizing antibodies in mice at levels comparable to those induced by inactivated polio vaccine. The addition of an aluminum hydroxide adjuvant to sVLP results in faster induction and a higher magnitude of neutralizing antibodies. Furthermore, our cryo-EM structural study of both sVLP and wtVLP reveals a native conformation for the sVLP and a non-native expanded conformation for the wtVLP. Our work not only validates the yeast-produced PV2 sVLP as a promising vaccine candidate with high production potential but also sheds light on the structural mechanisms that underpin the assembly and immunogenicity of the PV2 sVLP. These findings may expedite the development of sVLP-based PV vaccines.

## 1. Introduction

Poliovirus (PV) belongs to the *Enterovirus* genus of the *Picornaviridae* family. It can cause poliomyelitis, a highly infectious disease that primarily affects children and can result in paralysis or even death [1]. The PV genome, approximately 7.5 kb in length, is a single-stranded, positive-sense RNA enclosed within a capsid protein shell [2,3,4]. This genome encodes a large polyprotein precursor that is initially cleaved into the structural protein P1 and the nonstructural proteins P2 and P3 [3]. The P1 is further cleaved by a viral protease to produce the capsid subunit proteins VP0, VP1, and VP3. VP0 then undergoes autocleavage to produced VP2 and VP4 [5,6,7]. PV derived from cell culture exists in two particle forms: the mature virion, which contains the viral RNA genome, and the empty capsid (EC), which lacks the viral RNA genome. These two forms are antigenically and immunogenically distinct [2,8]. The PV native mature virion displays the so-called D-antigen and elicits an effective immune protection, whereas the naturally occurring EC is predominantly associated with the C-antigen and is unable to induce a protective immune response [4,9,10]. However, upon heating, the PV mature virion may undergo a conformational change, resulting in the conversion of the D-antigen to the C-antigen. This change leads to a loss of protective immunogenicity [11,12].

PV comprises serotypes 1, 2, and 3 (termed PV1, PV2, and PV3, respectively). Each of these serotypes can cause poliomyelitis [1]. For each serotype, two types of PV vaccines have been developed and are widely used: the formaldehyde-inactivated polio vaccine (IPV) and the live attenuated oral vaccine (OPV) [1]. Each vaccine type has its own advantages and disadvantages in terms of manufacturing and application. OPV is relatively inexpensive, easy to administer, and capable of inducing both systemic and mucosal immunity. However, due to its genetic instability, OPVs, especially OPV2, can revert to a neurovirulent form in vaccine recipients. This reversion can lead to rare cases of vaccine-associated paralytic poliomyelitis (VAPP) and the creation of circulating vaccine-derived poliovirus (cVDPV) strains [1,13]. On the other hand, IPV can provide protection against poliomyelitis in vaccinated individuals without the risk of virus reversion. However, it does not trigger mucosal immunity in the gut and therefore cannot prevent fecal-oral transmission of the virus within a population [14]. The production of both IPV and OPV requires the growth and handling of a large amount of live infectious PV, which carries the risk of accidental release [15]. Therefore, it is of significant importance to develop new-generation PV vaccines that do not involve the use of live virus in their production.

Virus-like particles (VLPs), produced in recombinant expression systems, are structurally similar to the authentic infectious virus but lack the infectious viral genome. They have been demonstrated to be a safe and effective vaccine platform for some viruses, such as human papilloma virus [16,17]. Previous work has shown that VLPs derived from the wildtype strain of PV (hereafter referred to as wtVLPs) can be generated in recombinant expression systems such as yeast and insect cells [18,19]. However, similar to the naturally occurring ECs, these VLPs are predominantly C-antigenic and therefore poorly immunogenic [18,20]. In 2017, Fox et al. identified stabilizing mutations on the capsid subunit proteins of all three PV serotypes by selecting virus mutants under increasing temperatures. These mutations rendered the corresponding mutant ECs highly thermostable and at least as immunogenic as the IPV counterparts [21]. This suggests that these mutations could be used to produce thermally stabilized VLPs (hereafter referred to as sVLPs) with desirable immunogenicity and protective efficacy. Indeed, recombinant sVLPs for PV3 have recently been produced in plant, yeast, insect, or mammalian cell expression systems and demonstrated to be thermostable and immunogenic [22,23,24,25]. These PV3 sVLPs, like the mature virion, adopt the native D-antigenic conformation [22,24,25], whereas PV3 wtVLPs are predominantly in the C-antigenic form [23,24,25]. For PV1 and PV2, the corresponding sVLPs could be recombinantly produced in insect cells [23]; however, their yields were unsatisfactory. Moreover, high-resolution structures of PV1 sVLP or PV2 sVLP remain unavailable thus far.

In this study, we demonstrate that both wtVLP and sVLP for PV2 can be efficiently produced in *Pichia pastoris* yeast through a novel expression strategy—directly co-expressing VP0, VP1, and VP3 capsid subunit proteins. We conducted a parallel comparison of the yeast-produced PV2 wtVLP and sVLP of their antigenicity, thermostability, and immunogenicity. Furthermore, we determined the structures of both wtVLP and sVLP by performing cryo-electron microscopy (cryo-EM) single particle analysis. The results not only demonstrate that the yeast-produced PV2 sVLP is a promising vaccine candidate with high-yield production capacity, but also reveal the structural mechanisms underlying the assembly and immunogenicity of PV2 sVLP. These findings may have important implications for the development of sVLP-based recombinant PV vaccines.

## 2. Methods

### 2.1. Cells

The 293T cells, obtained from the National Collection of Authenticated Cell Cultures (Shanghai, China), were maintained in Dulbecco’s modified Eagle’s medium (DMEM) containing 10% fetal bovine serum (FBS) and 100 U/mL penicillin-streptomycin (Gibco, Grand Island, NY, USA). The cells were grown at 37 °C under a 5% CO_2_ atmosphere. *PichiaPink*™ yeast strains (Invitrogen, Carlsbad, CA, USA) were cultivated following the manufacturer’s protocol.

### 2.2. Antibodies

Monoclonal antibody (mAb) 1050 was obtained from the National Institute for Biological Standards and Control (NIBSC, Hertfordshire, UK). Polyclonal antibodies against VP2, VP3 or VP1 were produced in-house by immunizing rabbits with recombinant VP2, VP3 or VP1 proteins from PV2, expressed in *Escherichia coli*, as described previously [26]. Additionally, polyclonal antibodies against PV2 VLP were generated by immunizing rabbits with PV2 wtVLP combined with Freund’s adjuvant (Sigma, St. Louis, MO, USA). The mouse mAb 1G3 was generated in-house from a PV2 sVLP-immunized mouse by using the conventional hybridoma technology as described previously [27]. Horseradish peroxidase (HRP)-conjugated 1G3 was prepared with the EZ-LinK^TM^ Plus Activated Peroxidase Kit (Pierce, Rickford, IL, USA) according to the manufacturer’s instructions.

### 2.3. Construction of Yeast Expression Vectors

The P1 coding sequences for PV2 wildtype strain MEF-1 (Gene Bank: AY238473.1) and the MEF-SC5a mutant were optimized for yeast expression and synthesized by Genscript (Nanjing, China). The VP0, VP1 and VP3 fragments of the optimized PV2 wtP1 gene were amplified by PCR and cloned into pPinK-HC vector (Invitrogen) using ClonExpress Ultra One Step Cloning kit (Vazyme, Nanjing, China), yielding plasmids HC-wtVP0, HC-wtVP1, and HC-wtVP3, respectively. The wtVP3 and wtVP0 expression cassettes were released from HC-wtVP0 and HC-wtVP3 by BamH I and Bgl II digestion, respectively, and then sequentially inserted into HC-wtVP1 from Bgl II site, yielding plasmid HC-wtVP^031^ that contained all three (wtVP0, wtVP3, wtVP1) expression cassettes. In the same way, plasmid HC-sVP^031^ that contained sVP0, sVP3 and sVP1 expression cassettes was constructed.

### 2.4. Pichia Pastoris Transformation and Selection of High-Expression Clones

Prior to *P. pastoris* transformation, plasmids HC-wtVP^031^ and HC-sVP^031^ were each linearized by digestion with *Afl* II. PichiaPink™ Strain 1 (Invitrogen) was transformed with the linearized plasmids and subsequently plated onto PAD plates as previously described [28]. To screen high-expression recombinant strains, individual colonies were randomly picked from the plates and small-scale expression experiments were performed according to the manufacturer’s instruction (Invitrogen). Induced yeast cells were harvested and lysed. Total soluble protein (TSP) in the clarified lysates were measured with Coomassie Plus Protein Assay Reagent (Pierce). VLP expression in the clarified lysates was determined by ELISA and Western blotting as described below. Briefly, lysate containing 25 µg TSP was added to wells of the 96-well ELISA plates and then incubated at 4 °C overnight; after three washes with PBST buffer, wells were blocked with 5% milk diluted in PBST for 1 h; after three washes, mAb1050 (diluted 1:1000 in 2% milk/PBST) was added and incubated at 37 °C for 2 h; after three washes, 50 µL of HRP-conjugated goat anti-mouse IgG (Sigma, Saint Louis, MO, USA) was added to each well and incubated at 37 °C for 1 h; after 5 washes, color was developed with TMB substrate (Pierce), and the absorbance at 450 nm was measured.

### 2.5. Preparation of VLP Antigens

For VLP antigen preparation, the identified high-expression yeast strains were cultivated and induced with methanol, following the manufacturer’s protocol (Invitrogen). After induction, the yeast cells were harvested by centrifugation and resuspended in 0.15 M phosphate buffered saline (PBS) buffer. The cells were then lysed using a high-pressure cell disrupter (JNBIO, Guangzhou, China) at 1800 Bar, and the resulting crude lysates were clarified by centrifugation at 12,000 rpm for 15 min. NaCl and PEG 8000 were then added to achieve final concentrations of 200 mM and 10% (*w*/*v*), respectively. The mixtures were stirred gently at 4 °C overnight to allow protein precipitation. The following day, the mixtures were centrifuged again at 12,000 rpm for 15 min; the resulting pellets were resuspended in PBS and subjected to a second round of centrifugation at 12,000 rpm for 15 min to remove insoluble materials. The clarified supernatants were ultracentrifuged at 27,000 rpm for 4 h over a 20% sucrose cushion. The obtained pellets were resuspended in PBS and layered onto 10–50% sucrose gradients for further ultracentrifugation at 39,000 rpm for 3 h. Twelve fractions were collected from top to bottom and analyzed. VLP-rich fractions, identified by SDS-PAGE and Western blotting, were pooled and pelleted through ultracentrifuging on a 20% sucrose cushion. The final VLP pellets were resuspended in PBS, quantified using the Bradford assay, and stored at −80 °C until further use.

### 2.6. SDS-PAGE and Western Blot Assays

Protein samples were subjected to 12% polyacrylamide gel electrophoresis and visualized with Coomassie blue staining. For Western blot analysis, proteins were transferred from SDS-PAGE gels to PVDF membranes. These membranes were then incubated with an antigen-specific primary antibody, followed by a matching HRP-conjugated secondary antibody. Western blots were developed using BeyoECL Plus kit (Beyotime, Shanghai, China) and digitally imaged.

### 2.7. Negative Stain Electron Microscopy

Purified VLPs were negatively stained using 0.5% aqueous uranyl acetate. Transmission electron microscopy was then conducted with a Tecnai G2 Spirit microscope operating at 120 KV.

### 2.8. ELISA

The antigenicity of VLPs was evaluated using indirect ELISAs. Protein samples were coated onto 96-well microtiter plates and incubated overnight at 4 °C. After washing with PBST buffer, the plates were blocked with 5% milk in PBST at room temperature (20–22 °C) for 1 h. Primary antibody in PBST with 1% milk was then applied and incubated at room temperature for 2 h. Following this, 50 µL/well of a corresponding HRP-conjugated secondary antibody in PBST with 1% milk was added and incubated at room temperature for 1 h. For color development, TMB substrate was applied, and the reaction was stopped with 1 N H_3_PO_4_ after 5–10 min. Absorbance was measured at 450 nm using a 96-well plate reader.

D antigen content in protein samples was quantitated by a non-competitive sandwich ELISA. Briefly, wells of 96-well ELISA plates were coated with 50 µL of D-antigen-specific mAb1050 (diluted 1:500 in PBS) at 4 °C overnight; after three washes with PBST buffer, plates were blocked with 5% milk diluted in PBST for 1 h; after three washes, protein samples to be tested or the international IPV standard (NIBSC code: 12-104) diluted in 2% milk/PBST were added and incubated at room temperature for 2 h; then, HRP-conjugated 1G3 in 2% milk/PBST was added and incubated at room temperature for 2 h. Color development and absorbance measurement were carried out as described above. The D-antigen content for each test sample was evaluated against the IPV standard with assigned D-antigen units.

### 2.9. Thermostability Analysis

For the temperature escalation experiment, aliquots of 100 ng of PV2 wtVLP or sVLP were incubated for 10 min at a range of temperatures from 30 °C to 60 °C and then cooled on ice. Then, the samples were analyzed by ELISA as described above.

For the time extension experiment, aliquots of 100 ng of PV2 wtVLP or sVLP were incubated at 37 °C for different periods (0 min, 10 min, 30 min, 1 h, 4 h, 8 h, 12 h, or 24 h) and then cooled on ice. Then, the samples were analyzed by ELISA as described above.

### 2.10. Mouse Immunization Study

The animal studies were approved by the Institutional Animal Care and Use Committee at Youshu Life Technology (Shanghai, China). Balb/c mice used in this study were purchased from Laboratory Animal Center of Shanghai Lingchang Biotechnology. The mice were kept in the specific pathogen-free animal facility of Youshu Life Technology with controlled temperature (20–26 °C), humidity (40–70%), and lighting conditions (12 h light/12 h dark cycle).

Prior to immunization, antigens were diluted with PBS or mixed with aluminum hydroxide adjuvant (Shanghai Bovax Biotechnology, Shanghai, China) to make the experimental vaccines. A single injection dose contained 0.5 or 1 µg of PV2 wtVLP or sVLP without aluminum hydroxide, or 0.5 µg of PV2 sVLP plus 500 µg aluminum hydroxide, or half human dose of commercial IPV vaccine (4 DU for PV2, Sanofi Pasteur, Lyon, France) in a final volume of 500 µL, respectively.

Groups of 9–10 female Balb/c mice (6–8 week) were injected intraperitoneally (i.p.) with one of the experimental vaccines at week 0, 2 and 4. Blood samples were collected at week 4 and week 6. The resulting mouse sera were incubated at 56 °C for 30 min to inactivate the complement and then stored at −80 °C until use.

### 2.11. Preparation of PV2 Pseudovirus

PV2 pseudovirus was generated by co-transfecting 293T cells with three plasmids, including P1-expressing plasmid, replicon plasmid, and T7 polymerase-expressing plasmid. The PV2/Sabin2 capsid protein gene P1 (GenBanck: 184220) linking with the dsRed gene via a 2A cleavage signal sequence were synthesized by Genscript (Nanjing, China) and cloned into pCDNA3.3 (Invitrogen), generating the P1-expressing plasmid designated pcDNA3.3-dsRed/Sabin2-P1 (Supplementary Figure S2). The DNA sequence of the PV1/Mahoney genome (GenBanck: V01149) with its P1 gene replaced by eGFP was synthesized by Genscript and cloned downstream the T7 promoter, yielding the RNA replicon vector designated pT7-Replicon-eGFP. The T7 polymerase (GenBanck: M38308) was also synthesized by Genscript and cloned into pcDNA3.3, yielding the T7 polymerase-expressing plasmid pcDNA3.3-T7.

For pseudovirus production, 293T cells were seeded into 6-well plates at a density of 5 × 10^5^ cells/well and incubated at 37 °C with 5% CO_2_ for 24 h. Cells at 60% confluency were co-transfected with 2 μg pcDNA3.3-dsRed/Sabin2-P1, 1 μg pcDNA3.3-T7 and 1 μg pT7-Replicon-eGFP formulated with Lipo3000 transfection reagent (Invitrogen). Protein expression was monitored under a fluorescence microscope at 48 h post transfection. Cell cultures were collected and subjected to three freeze/thaw cycles. Cellular debris was removed by centrifugation at 1700× *g* for 5 min. The resulting supernatant was passed through a 0.22 μm filter, yielding final pseudovirus stocks.

For pseudovirus titration, the pseudovirus stocks were serially diluted by ten-fold and added to wells (100 μL/well) of 96-well plates. Then, 100 μL of suspended 293T cells (4 × 10^4^ cells) was added to each well and incubated at 37 °C with 5% CO_2_ for 20 h. After gently removing the medium, cells in the wells were analyzed by ImmunoSpotS6 (CTL) and the number of GFP fluorescent spots was counted. Pseudovirus titer was calculated as GFP fluorescent focus unit (FFU) per mL of the pseudovirus stock.

### 2.12. Pseudovirus-Based Neutralization Assay

Pseudovirus neutralization assay was conducted in a 96-well plate. Briefly, 50 μL of 2-fold serially diluted serum sample were mixed with an equal volume of pseudovirus containing 200 FFU, followed by incubation at 37 °C for 2 h. Then, 4 × 10^4^ 293T cell were added to each well of the 96-well plate and incubated at 37 °C with 5% CO_2_. After 20 h, the medium was gently removed, and the cells were examined for GFP fluorescent spots using the ImmunoSpotS6 plate reader. The neutralization titers were defined as the highest serum dilutions that inhibited at least 90% of GFP fluorescent focus formation.

### 2.13. Cryo-EM Sample Preparation and Data Collection

A 2.4 µL aliquot of the purified wtVLP and sVLP samples (prepared in Section 2.5) was applied onto a plasma-cleaned holey carbon grid (R2/1, Cu, 200 mesh; Quantifoil), respectively. The grids were blotted using Vitrobot Mark IV (Thermo Fisher Scientific, Waltham, MA, USA) with a blot force of −1 and a blot time of 1 s at 100% humidity and 8 °C. Afterwards, the blotted grids were plunged into liquid ethane cooled by liquid nitrogen.

Cryo-EM movies were recorded using K3 directed electron detector (Gatan, Pleasanton, CA, USA) on a Titan Krios electron microscope (Thermo Fisher Scientific) operating at 300 kV. The movies for the two datasets were recorded at a magnification of 64,000× in counting mode (corresponding to a pixel size of 1.093 Å). Each frame was exposed for 0.1 s, with a total accumulation time of 3 s, resulting in a total accumulated dose of 50.2 e^−^/Å^2^ on the specimen.

### 2.14. Cryo-EM Single Particle 3D Reconstruction

Data processing procedures for both wtVLP and sVLP datasets were similar. For each dataset, the motion correction of the image stack was performed using the Motioncor2 module embedded in Relion 3.1 [29,30], and contrast transfer function (CTF) parameters were determined using CTFFIND4 [31] prior to further data processing. In the case of wtVLP, 136,695 particles were autopicked from 1550 micrographs by crYOLO [32], and 107,781 remained after reference-free 2D classification. These particles were refined using the PV3 VLP map (EMD-3747) as the initial model [22]. We then performed 3D auto-refinement with icosahedral symmetry, using the previous refinement result as a reference. After CTF refinement, Bayesian polishing, and further refinement, a wtVLP map was obtained at a resolution of 3.1 Å. For sVLP, automated particle picking by Relion resulted in 13,227 particles from 1189 micrographs, and 9860 particles were selected after reference-free 2D classification. Following a similar process of refinement, CTF refinement, Bayesian polishing, and further refinement, a 3.1 Å sVLP map was acquired. The overall resolutions for the cryo-EM maps were determined based on the gold-standard criterion, using a Fourier shell correlation (FSC) of 0.143 [33].

### 2.15. Atomic Model Building and Analysis

We utilized the PV1 135S-like expanded particle model (PDB: 6P9O) as template to build the homology model of wtVLP via the SWISS-MODEL webserver [34,35]. For the sVLP, we employed the corresponding cryo-EM structures of PV2 (PDB: 8ayz) from a previous study as initial model [36]. We then used Rossetta [37] to refine the model adaptively against the relevant cryo-EM map. The clear side chain densities present in our maps allow for comprehensive adjustments and refinements of the atomic model using COOT [38]. Further refinement was achieved using the phenix.real_space_refine module in Phenix [39]. The final atomic models were validated using phenix.molprobity [40]. We analyzed the interaction interface through the PISA server [41].

For visual representation and analysis of hydrophobic interactions, we utilized UCSF Chimera and ChimeraX [42,43].

### 2.16. Statistical Analysis

All statistical analysis was performed using GraphPad Prism version 8. Neutralizing titers of the mouse sera from different immunization groups were compared by Mann–Whitney two-tailed test. Statistical significance between groups was indicated as follows: ns (no significant difference), *p* ≥ 0.05; *, *p* < 0.05; **, *p* < 0.01; ***, *p* < 0.001; ****, *p* < 0.0001.

## 3. Results

### 3.1. Expression and Assembly of PV2 wtVLP and sVLP in Pichia Pastoris

**Figure 1:**
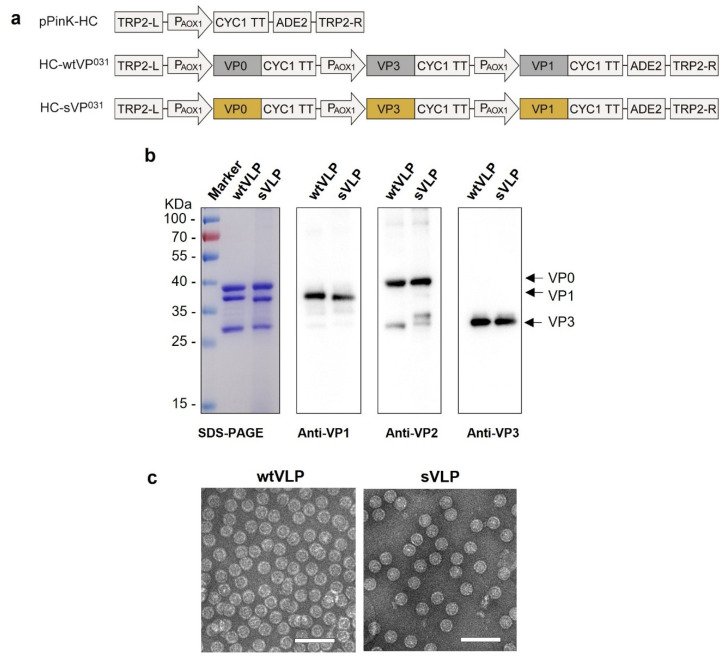
Expression of PV2 wtVLP and sVLP in *Pichia pastoris*. (**a**) Diagrams of the expression vectors. TRP2-L and TRP2-R, the up- and down-stream parts of the TRP region; P_AOX1_, AOX1 promoter; CYC1 TT, CYC1 transcription termination region; ADE2, expression cassette encoding phosphoribosylaminoimidazole carboxylase, used as the selection marker. (**b**) SDS-PAGE and Western blot analysis of the purified wtVLP and sVLP. The primary antibodies used in the Western blot assays are indicated. (**c**) Visualization of *P. pastoris*-derived VLPs by negative stain electron microscopy. Bar = 100 nm.

Previous studies have shown that co-expression of P1 and 3CD of PV3 in recombinant systems resulted in cleavage of P1 by 3CD to yield three capsid subunit proteins, VP0, VP1, and VP3, all of which co-assembled into PV3 VLPs [22,24,25]. Therefore, we hypothesized that direct simultaneous expression of VP0, VP3, and VP1 proteins of PV2 in yeast may lead to more efficient VLP assembly. Hence, we constructed two vectors (termed HC-wtVP^031^ and HC-sVP^031^), each of which contains three expression cassettes for VP0, VP3, and VP1 of the PV2 wildtype strain (MEF-1) or the MEF-SC5a mutant [21], respectively (Figure 1a). The vectors were individually transformed into the PichiaPink™ yeast and the resulting transformants were screened for PV2 antigen content by ELISA using the D-antigen-specific monoclonal antibody, mAb 1050. After the high-expressing *P. pastoris* clones for both HC-wtVP^031^ and HC-sVP^031^ were identified, they were cultured, induced with methanol and subsequently subjected to a purification process as described in the Methods Section. SDS-PAGE and Western blotting analyses of the purified materials showed three protein bands for both HC-wtVP^031^- and HC-sVP^031^-transformed yeast, including a ~40 KDa band, a ~37 KDa band and a ~29 KDa band which were recognized by the VP0-specific, the VP1-specific, and the VP3-specific polyclonal antibodies, respectively (Figure 1b), indicating the successful co-expression and assembly of VP0, VP3 and VP1. Examination of the purified samples by electron microscopy revealed the presence of spherical particles with a diameter of 30 nm (Figure 1c). Collectively, these data demonstrate the VP0, VP3 and VP1 subunit proteins expressed in the HC-wtVP^031^- and HC-sVP^031^-transformed yeast were co-assembled into wtVLP and sVLP, respectively. By using our current expression and purification protocols, we routinely obtained around 0.2 milligram of purified VLP per gram of yeast wet weight.

### 3.2. Antigenicity Analysis of Yeast-Produced PV2 wtVLP and sVLP

**Figure 2:**
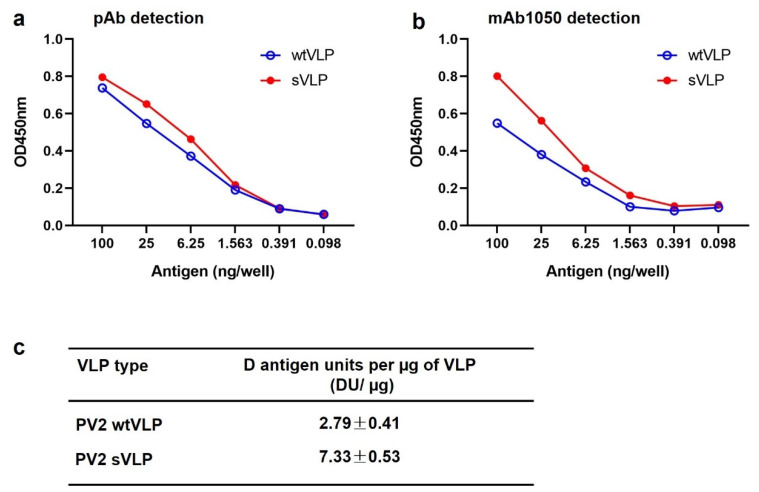
Antigenicity of yeast-produced PV2 wtVLP and sVLP. (**a**) Reactivity of wtVLP and sVLP with anti-PV2 polyclonal antibody in ELISA. (**b**) Reactivity of wtVLP and sVLP with the D-antigen-specific mAb 1050 in ELISA. (**c**) D-antigen levels in the wtVLP or sVLP preparations. The data are presented as means ± SD from three independent measurements.

Native PV particles express two distinct antigens, including immunogenic D-antigen and non-immunogenic C-antigen [2]. We assessed the antigenicity of the yeast-produced PV2 wtVLP and sVLP by ELISA with an anti-PV2 polyclonal antibody and the D-antigen-specific mAb 1050. As shown in Figure 2a,b, both sVLP and wtVLP reacted similarly to the anti-PV2 sVLP polyclonal antibody, whereas the sVLP sample exhibited much higher reactivity to the mAb 1050 than did the wtVLP. These data indicate that the sVLP expresses much more D-antigen than the wtVLP. The C-antigen levels were not assessed due to lack of appropriate antibody. We further quantitated D-antigen in the sVLP and wtVLP preparations by sandwich ELISA with the international IPV standard (NIBSC code: 12/104) as reference (Supplementary Figure S1). The results showed that 1 µg of PV2 sVLP contained 7.33 units of D-antigen whereas only 2.79 units of D-antigen were present in 1 µg of PV2 wtVLP (Figure 2c).

### 3.3. Distinct Thermostability for PV2 wtVLP and sVLP

**Figure 3:**
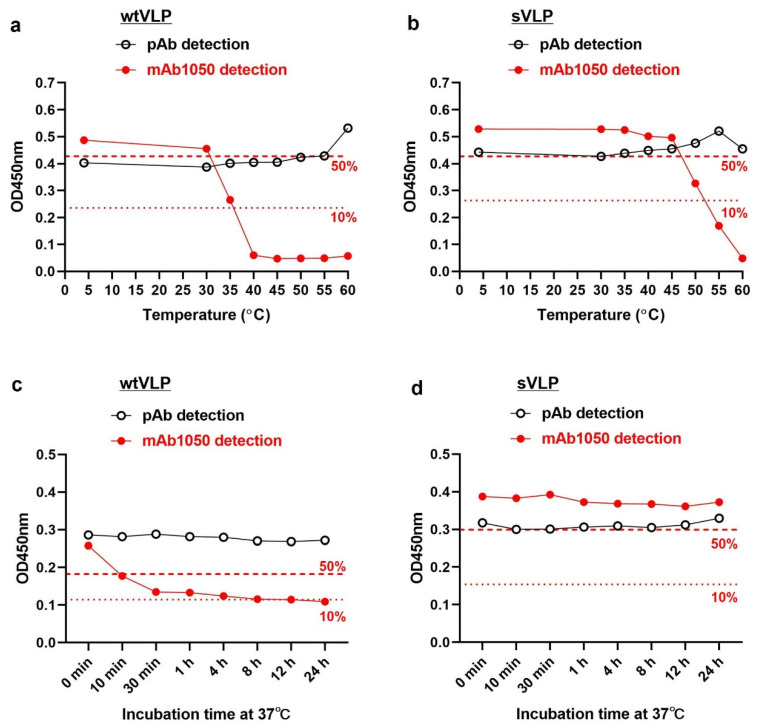
Thermostability of PV2 wtVLP and sVLP. (**a**,**b**) Equal amounts of (**a**) wtVLP and (**b**) sVLP were subjected to incubation at different temperatures for 10 min and then analyzed by ELISA using the pAb or mAb1050. The red dash line and the dotted line indicate the mAb1050 reactivity (OD450 nm values) corresponding to 50% and 10% of the D-antigen in the untreated VLPs, respectively. Representative data from two independent experiments are shown. (**c**,**d**) Equal amounts of (**c**) wtVLP and (**d**) sVLP were subjected to incubation at 37 °C for different time periods as indicated and then analyzed by ELISA using pAb or mAb1050. The red dash line and the dotted line indicate the mAb1050 reactivity (OD450 nm values) corresponding to 50% and 10% of the D-antigen in the untreated VLPs, respectively. Representative data from two independent experiments are shown.

For thermostability assessment, PV2 wtVLP and sVLP samples were heated for 10 min at a range of temperatures from 30 °C to 60 °C and then analyzed by ELISA. VLP samples stored at 4 °C were also included in the assay, serving as a positive control. As shown in Figure 3a,b, for both wtVLP and sVLP, their reactivity to the anti-PV2 polyclonal antibody, which represents the levels of total antigen, was roughly unchanged regardless of treatment temperature. The D-antigen content (reflected by the binding activity to the mAb 1050) in the wtVLP dropped drastically at 35 °C and was almost completely lost at 40 °C, whereas the sVLP exhibited significantly improved D-antigenic thermostability, maintaining >50% D-antigen content even at 45 °C. Next, we treated wtVLP and sVLP at 37 °C for different periods of time and then evaluated total antigen and D-antigen content. For the wtVLP, its D-antigen content reduced by more than 50% upon incubation at 37 °C for 10 min and by approximately 90% after 30 min; in sharp contrast, the sVLP retained similar levels of D-antigen throughout the 24 h course (Figure 3c,d). Collectively, these data demonstrate that PV2 sVLP is superior to wtVLP in terms of D-antigen thermostability.

### 3.4. PV2 sVLP, but Not wtVLP, Potently Induces Neutralizing Antibody Response in Mice

**Figure 4:**
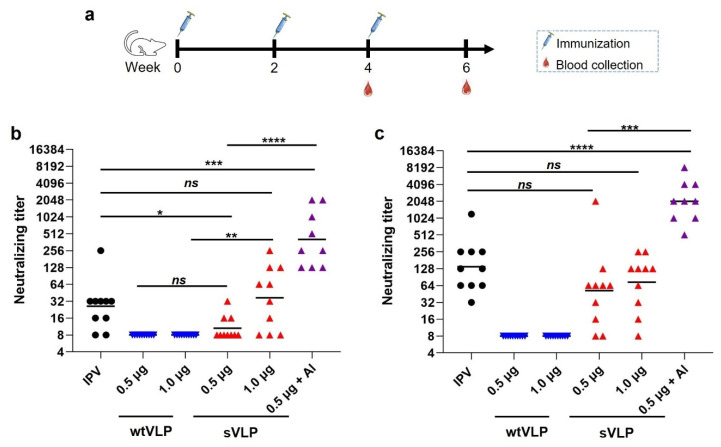
Immunogenicity of PV2 wtVLP and sVLP in mice. (**a**) Mouse immunization and sampling schedule. (**b**) Neutralizing titers of the week-4 antisera against PV2 pseudovirus. (**c**) Neutralizing titers of the week-6 antisera against PV2 pseudovirus. Serum samples that exhibited less than 90% neutralization at the lowest serum dilution (1:16) were assigned a titer of 8 for computation of geometric means. Each symbol represents one mouse. The geometric mean titer for each group is shown. Statistical significance between two groups was calculated by Mann–Whitney two-tailed test. ns, no significant difference (*p* ≥ 0.05; *, *p* < 0.05; **, *p* < 0.01; ***, *p* < 0.001; ****, *p* < 0.0001.

To evaluate the immunogenicity of PV2 wtVLP and sVLP, we performed mouse immunization experiments. Groups of Balb/c mice (*n* = 10) were immunized with different doses (0.5 or 1 µg) of the wtVLP or sVLP, or commercial IPV (containing 4 units of PV2 D-antigen, corresponding to half of a single human dose) that served as the positive control. An additional group of mice (*n* = 9) was administered 0.5 µg sVLP formulated with aluminum hydroxide adjuvant. Sera were collected from individual mice after the second and the third doses and analyzed for their neutralization potency (Figure 4a). Due to the restricted access to live PV2, we generated GFP-expressing PV2 pseudovirus and used it to perform neutralization assays (Supplementary Figure S2). As shown in Figure 4b,c, neither the week-4 nor the week-6 sera from the wtVLP-immunized mice exhibited any neutralization even at the lowest serum dilution (1:16) tested; in contrast, 30% and 70% of the mice in the 0.5 µg and 1.0 µg sVLP groups, respectively, were already sero-converted at week 4, and both the number of responder mice and neutralizing titers increased after the third immunization with geometric means being 52, and 74 for the 0.5 µg and 1.0 µg sVLP groups, respectively, at week 6. These results demonstrate that the sVLP, but not the wtVLP, could potently elicit neutralizing antibodies. As expected, the mice immunized with IPV (0.5 human dose) also produced neutralizing antibodies. The neutralizing antibody levels induced by the IPV were comparable to those induced by 1.0 µg sVLP at weeks 4 and 6 (Figure 4b,c). Notably, it was found that the mice immunized with 0.5 µg sVLP formulated with aluminum hydroxide adjuvant (the “0.5 µg sVLP + Al” group) produced significantly higher titers of neutralizing antibodies than those immunized with either IPV or 0.5 µg sVLP alone (Figure 4b,c), indicating a potent immune boosting effect of the adjuvant.

### 3.5. Cryo-EM of PV2 wtVLP and sVLP Reveals sVLP Adopting a Native Conformation

**Figure 5:**
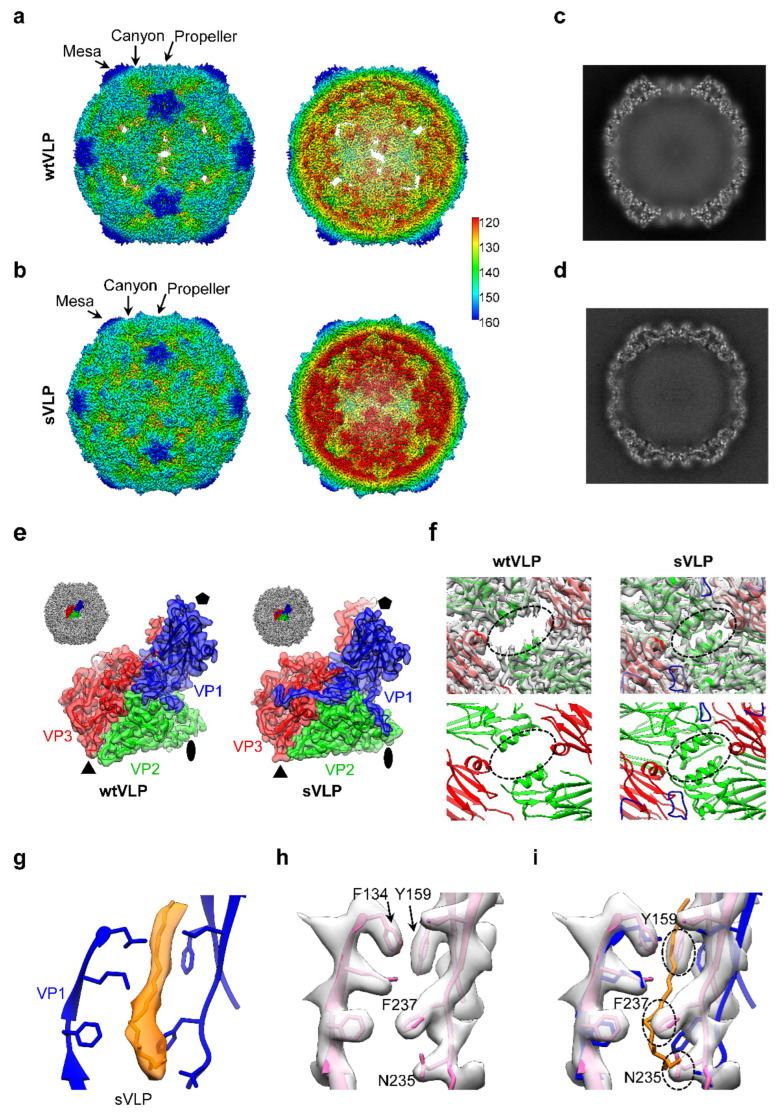
Cryo-EM structures of the wtVLP and sVLP reveal that sVLP adopts a native conformation. (**a**,**b**) The overall cryo-EM density map of (**a**) wtVLP and (**b**) sVLP, viewed along the two-fold axis. The color bar indicates the corresponding radius from the center of the particle (units in Å). (**c**,**d**) The central section of the cryo-EM map of (**c**) wtVLP and (**d**) sVLP, illustrating the empty interior. (**e**) Model and map fitting of a single asymmetric unit of our cryo-EM maps. VP1, VP2, and VP3 are colored in blue, green, and red, respectively. (**f**) Zoom-in view of the VLPs around the 2-fold symmetry axis. (**g**) The density of the “pocket factor” in the sVLP pocket, modeled as sphingosine (in orange). (**h**,**i**) The “pocket” of the wtVLP map. The blue sVLP model with pocket factor colored in orange fitted in the wtVLP map shows the clash of pocket factor with Y159, F237 and N235 (**i**).

To elucidate the molecular basis underlying the distinct antigenicity, thermostability, and immunogenicity between PV2 wtVLP and sVLP, we performed cryo-EM single particle analysis on these two VLPs (Supplementary Figure S3a,b and Supplementary Table S1). The cryo-EM density maps for both wtVLP and sVLP were determined at 3.1 Å resolution (Figure 5a,b, and Supplementary Figure S3). The VLP maps reveal a classic icosahedral configuration characteristic of enteroviruses, marked by distinctive surface elements. These elements comprise a “mesa” at the five-fold-symmetry axes, an encircling “canyon” around the mesa, and a “tripod” feature resembling a three-bladed propeller at the three-fold axes (Figure 5a,b). However, the wtVLP appears to be in an expanded state compared to the sVLP, as evidenced by the radius of the wtVLP being 170 Å and that of the sVLP being 163 Å (Figure 5a,b). In addition, there is an open channel at the two-fold axes in wtVLP, whereas the channel is closed in the sVLP (Figure 5a,b,f). Notably, no density corresponding to RNA was present inside the capsid shell for either VLPs (Figure 5c,d).

We then built atomic models for each map. The model fits in the corresponding map very well and displays high-resolution structural features (Figure 5e and Supplementary Figure S3e). Structural comparison revealed that the two-fold axis channel, formed by VP2 and VP3, is markedly opened in the wtVLP, whereas it is closed in the sVLP (Figure 5f). Noteworthy, a lipid “pocket factor” modeled as sphingosine was observed within the VP1 “pocket” of the sVLP (Figure 5g), while the “pocket” in the wtVLP was empty (Figure 5h). Detailed structural analysis shows that in the wtVLP, the pocket factor binding site is preoccupied by the side chains of Y159, F237, and N235 (Figure 5i), and the hydrophobic pocket collapsed, with its volume reducing from 914 Å^3^ to 577 Å^3^ (measured by ProteinsPlus [44]). As a result, the wtVLP VP1 “pocket” can no longer accommodate lipids. For enteroviruses, their mature virions are in a compact state and typically harbor a lipid pocket factor in the hydrophobic VP1 pocket, while their ECs are relatively expanded and lack a pocket factor [2,45]. We compared our VLP structures with the PV2 mature virion structure (PDB: 1EAH [46] and PDB:8E8S [47]) and the PV1 expanded 135S particle structure (PDB: 6P9O [34]). The structural comparison showed that the wtVLP is highly similar to the PV1 135S-like particles, and the sVLP structure aligns well with the native PV2 model (Supplementary Figure S3f). These data indicate that the PV2 sVLP adopts a native conformation similar to that of the corresponding mature virion, whereas the PV2 wtVLP is in a non-native expanded state.

### 3.6. The sVLP Is More Structurally Stable Than the wtVLP

**Figure 6:**
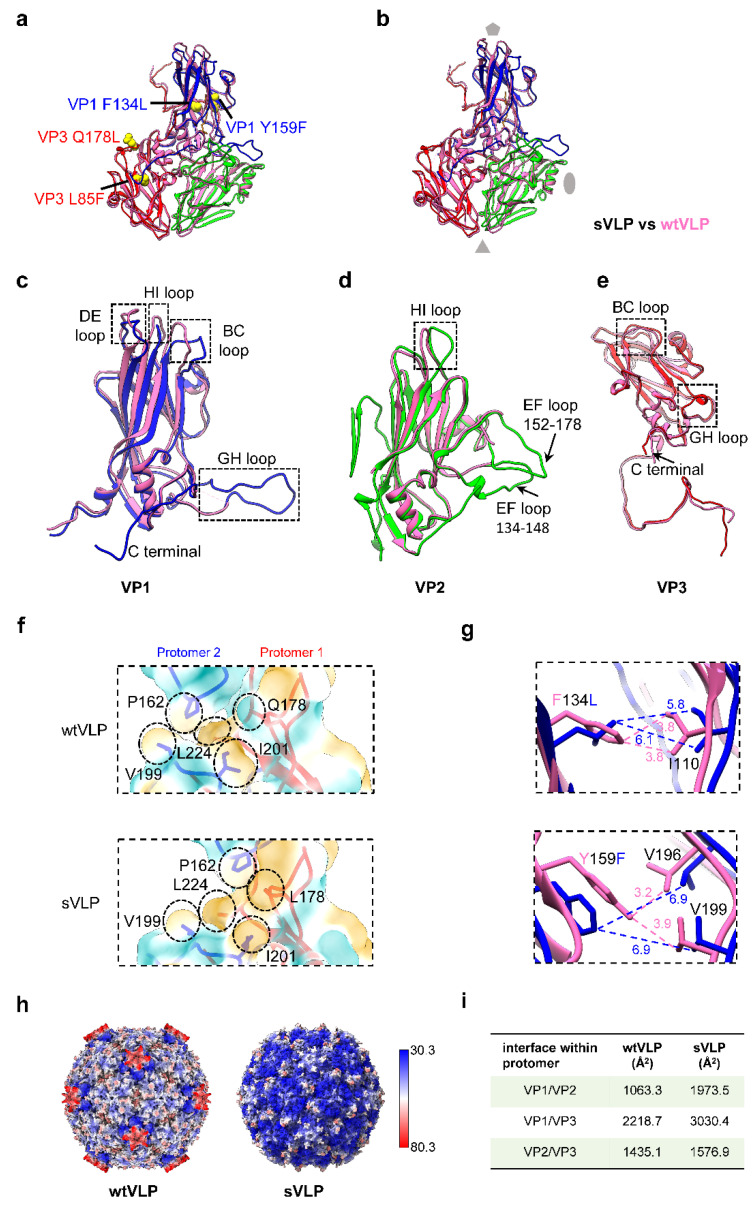
Structural comparison and mutation site analysis of the VLPs. (**a**) Model of the asymmetric unit for sVLP, with mutations shown as yellow spheres. (**b**) A comparison of the asymmetric units of wtVLP (in hot pink) and sVLP (in colors). (**c**–**e**) Superposition of individual proteins: VP1 (**c**), VP2 (**d**), and VP3 (**e**) between wtVLP (in hot pink) and sVLP. (**f**) Surface representation of the amino acids surrounding Q178 (wtVLP)/L178 (sVLP), colored by hydrophobicity from dark cyan (hydrophilic) to dark gold (hydrophobic). The neighboring amino acids are labelled. (**g**) Measurements of the distances between surrounding amino acids of VP1 F134 (wtVLP),/L134 (sVLP) and Y159 (wtVLP)/F159 (sVLP). The wtVLP VP1 colored in hot pink and sVLP in blue. (**h**) The B-factor display of the two VLPs. The B-factor was calculated utilizing ChimeraX, with blue representing a low B-factor and red representing a high B-factor. (**i**) The calculated VPs interface within protomer.

The structures of individual VP2 and VP3 proteins in both the wtVLP and sVLP are in general quite similar, each consisting of eight-stranded antiparallel β-barrel core (Figure 6a,b). However, key differences between wtVLP and sVLP are observed in several regions. Variations are present in the VP1 β-barrel pocket, as well as in loop regions, and the C terminus of the subunit proteins (Figure 6c–e). These differences include the DE, HI, and BC loops adjacent to the five-fold axis, the GH loop near the quasi-three-fold junction, and the C terminus of VP1. Furthermore, distinctions are observed in the EF loop of VP2 near the southern edge of the canyon, the HI loop near the three-fold axis, the GH loop near the quasi-three-fold junction, the BC loop at the VP3/VP2 interface, and the C terminal region at the VP3/VP1 interface of VP3 (Figure 6c–e). It is significant to highlight that these areas are all exposed on the surface and have been experimentally identified as antigenic epitopes [48,49]. While VP4 and the N-terminal (residues 1–68) of VP1 are unresolved in both VLPs, the sVLP, unlike the wtVLP, has ordered structures in the C-terminal of VP1, residues 213–231 in the VP1 GH loop, residues 134–149 and 160–173 in the VP2 EF loop, and the VP3 GH loop (Figure 6c–e). Moreover, the B-factor of the sVLP is generally lower than that of the wtVLP (Figure 6h). Collectively, the increase in the number of ordered parts of capsid proteins and lower B-factor suggest that the sVLP is more structurally stable than the wtVLP.

In comparison to the PV2 wtVLP, the sVLP contains five amino acid alterations, including VP1 T41I, VP1 F134L, VP1 Y159F, VP3 L85F, and VP3 Q178L (Figure 6a). These mutations are believed to be responsible for the stabilization of the PV2 empty capsid [21]. According to our structural analysis, VP1 F134L and Y159F are situated in the VP1 pocket, VP3 Q178L is located at the protomer interface and in proximity to the quasi-three-fold, L85F is present on the VP3 β-sheet, and VP1 T41I, which remains unresolved, is at the pentamer interface within the capsid (Figure 6a). The VP3 Q178 is surrounded by several hydrophobic amino acids that interact with the neighboring protomers. In the sVLP, the Q178L mutation transforms the residue from a hydrophilic to a hydrophobic amino acid, thereby enhancing the hydrophobic interaction between protomers (Figure 6f). The F134L and Y159F mutations in the sVLP VP1 pocket result in their displacement away from surrounding amino acids. Specifically, F134L extends the distance between itself and I110 from ~4 Å to ~6 Å, while Y159F increases the distance between itself and either V196 or V199 from ~3 Å to ~7 Å (Figure 6g). This could potentially lead to an enlarged pocket to accommodate the lipid pocket factor. Importantly, these mutations also induce compact interaction within the protomer (Figure 6i) and enlarged interface between subunit proteins in the pentamer, leading to a more stable and compact conformation. This allows for an increase in the interactions that hold the particle together (Supplementary Table S2).

## 4. Discussion

Genetically thermostabilized poliovirus empty capsids or sVLPs represent a promising strategy for the development of non-replicating PV vaccines [21,22,24,25]. To harness the potential of sVLP as a new-generation vaccine to replace current IPVs, it is critical to achieve high-yield production of sVLPs for all three PV serotypes using an economical and scalable recombinant system, and to conduct a thorough characterization of these sVLPs. In this study, we produced high levels of PV2 sVLP and wtVLP in *P. pastoris* yeast. We then conducted a comprehensive comparison of their antigenicity, thermostability, and immunogenicity. In addition, to the best of our knowledge, we determined the near-atomic-resolution cryo-EM structure of PV2 sVLP for the first time.

In this study, we employed a unique expression strategy—co-expression of VP0, VP3, and VP1—to produce PV2 wtVLP and sVLP in yeast. This strategy may offer several advantages over the P1/3CD co-expression approach that has been commonly used to produce VLPs of a number of enteroviruses, including PV3, in yeast [25,28,50,51,52]. Firstly, the P1/3CD co-expression approach relies on the cleavage of P1 by 3CD to produce the VP0, VP1, and VP3 subunit proteins, a process that can be somewhat inefficient or incomplete; in contrast, the VP0/VP1/VP3 co-expression strategy eliminates this cleavage step, potentially leading to more efficient VLP assembly. Secondly, our VP0/VP1/VP3 co-expression strategy circumvents the potential toxicity of 3CD, thereby promoting better yeast growth and accumulation of the target protein. Indeed, we found that the co-expressed VP0, VP3, and VP1 proteins of PV2 readily assembled into VLPs in *P. pastoris*. High-yield VLP production (up to 0.2 mg of purified VLP per gram of yeast wet weight) was achieved under our current laboratory setting. Importantly, the purified sVLP displayed protective D-antigen (7.33 DU per µg protein). That is to say, under our current setting, each gram of sVLP-expressing yeast can produce 1466 DU, equivalent to 183 human doses (8 DU/dose for PV2) of commercial IPV vaccine). We speculate that sVLP expression levels could be further increased through high-density fermentation and optimization of yeast culture and induction conditions. This high-yield sVLP production capacity and scalability of the yeast system may facilitate future mass vaccine production.

Our antigenicity analysis shows that the yeast-produced PV2 sVLP is predominantly D-antigenic, possessing 7.33 units of D-antigen per µg protein, approximately 2.6-fold higher than the D-antigen level in the wtVLP. Furthermore, the sVLP exhibits significantly greater thermostability than the wtVLP in terms of preserving their D-antigen content. Specifically, the sVLP retains over 50% of its D-antigen even after being heated for 10 min at temperatures up to 45 °C, whereas the wtVLP loses more than 50% of its D-antigen when exposed to temperatures as low as 35 °C (Figure 3). At 37 °C, the sVLP remains stable for at least 24 h, while the wtVLP loses more than 50% of its D-antigen within 10 min (Figure 3). Clearly, the PV2 sVLP demonstrates significantly improved D-antigenic thermostability compared with the wtVLP. Mouse immunization experiments showed that the yeast-produced PV2 sVLP is immunogenic, able to effectively induce neutralizing antibodies (Figure 4). Despite the yeast-derived wtVLP also possessing a reasonable amount of D-antigen, it fails to trigger a neutralizing antibody response in vivo (Figure 4), likely due to its poor thermostability especially at 37 °C, the normal physiological temperature of mice and humans. We speculate that, once delivered into mouse or human bodies, the D-antigen presented on the wtVLP might rapidly convert to a non-D-antigenic conformation, thereby unable to trigger D-antigen-specific antibody responses. In contrast, the sVLP effectively preserves the D-antigenic conformation at 37 °C, allowing its D-antigen to be recognized and processed by the immune system in vivo, thereby stimulating an antibody response towards the protective D-antigen. Hence, our study not only verifies the immunogenicity of PV2 sVLP but also underscores D-antigenic thermostability (especially at 37 °C) as a critical parameter in the evaluation of PV vaccine candidates.

Previous research has demonstrated that the addition of aluminum hydroxide (alum), a widely used vaccine adjuvant, to IPV resulted in a ten-fold increase in neutralizing antibody titer in mice and rats compared to IPV alone [53]. This finding illustrates a significant dose-sparing effect. In our current study, we found that formulation with an alum adjuvant could significantly enhance the immunogenicity of the yeast-derived PV2 sVLP in mice (Figure 4). Our data thus suggest that the sVLP–aluminum hydroxide formulation could serve as a more effective PV candidate vaccine. Such a vaccine formulation may allow for dose reduction, thereby increasing the availability of the vaccine and reducing its cost. This would be beneficial for mass immunization on a global scale.

It is generally recognized that the mature PV virion is associated with the immunogenic D-antigen, whilst naturally occurring EC is associated with the C-antigen and is non-immunogenic. Our cryo-EM analysis revealed that the PV2 sVLP closely resembles the native mature virion in the capsid region, whereas the PV2 wtVLP adopts an expanded conformation similar to the EC or 135S particle. Similar structural observations have been made for the corresponding non-immunogenic wtVLP and immunogenic sVLP of PV3 [22,24,25]. These findings collectively suggest that the antigenicity and immunogenicity of a PV vaccine candidate are imprinted in their structures. Our structural analysis shows that the “stabilizing” mutations on the PV2 sVLP result in an increased interaction interface area and a more favorable surface property compared with the wtVLP (Supplementary Table S2). This potentially allows the particle be held together more tightly. In addition, the residues F134L and Y159F enlarge the “pocket” to accommodate a lipidic pocket factor, which is known to stabilize the particle [2,45]. Indeed, a sphingosine-like pocket factor was observed in the PV2 sVLP but not in the PV2 wtVLP (Figure 5). Interestingly, the doorloop 232–236 in our PV2 sVLP was found to be in the “down” conformation whereas it adopts the “up” conformation in the PV2 native virion model (Supplementary Figure S3f). Such a “down” conformation of the doorloop 232–236 has been implicated to potentially prevent the pocket factor release [54], thereby enhancing particle stability. All of the above-mentioned structural modifications may contribute to the observed thermostabilization and immunogenicity of the PV2 sVLP.

In summary, our research provides compelling evidence that immunogenic PV2 sVLP can be produced at high levels in yeast—a cost-effective, highly scalable recombinant system; and the addition of an alum adjuvant to yeast-produced sVLP can greatly enhance vaccine potency. In addition, by acquiring and comparing the high-resolution structures of PV2 wtVLP and sVLP, we elucidate the structural mechanism that underpins the assembly and immunogenicity of PV2 sVLP. These findings should expedite the development of sVLP-based vaccines for PVs and other picornaviruses with significant clinical relevance.

## Data Availability

All data needed to evaluate the conclusions in the paper are present in the paper and the Supplementary Materials. Cryo-EM maps have been deposited in the Electron Microscopy Data Bank (http://www.emdataresource.org, accessed on 18 September 2024) with the accession numbers of EMD-60095 for wtVLP and EMD-39895 for sVLP. The associated models have been deposited to the Protein Data Bank (https://www.rcsb.org/, accessed on 18 September 2024) with accession codes 8ZH6 and 8ZB6.

## Supplementary Materials

The following supporting information can be downloaded at: https://www.mdpi.com/article/10.3390/vaccines12091077/s1, Figure S1: D antigen quantitation by sandwich ELISA; Figure S2: PV2 pseudovirus neutralization assay; Figure S3: Cryo-EM analysis of the VLPs; Table S1: Cryo-EM data collection and refinement statistics for wtVLP and sVLP; Table S2: Calculated interface area for the wtVLP and sVLP.
