# Supplementary material for: Vaccine Potency and Structure of Yeast-Produced Polio Type 2 Stabilized Virus-like Particles
Source: Vaccines (Basel). 2024 Sep 20;12(9):1077. doi: 10.3390/vaccines12091077 (PMC11435573; doi:10.3390/vaccines12091077)
Supplement: Supplementary file 1 [file vaccines-12-01077-s001.zip › vaccines-3178772-supplementary.pdf]

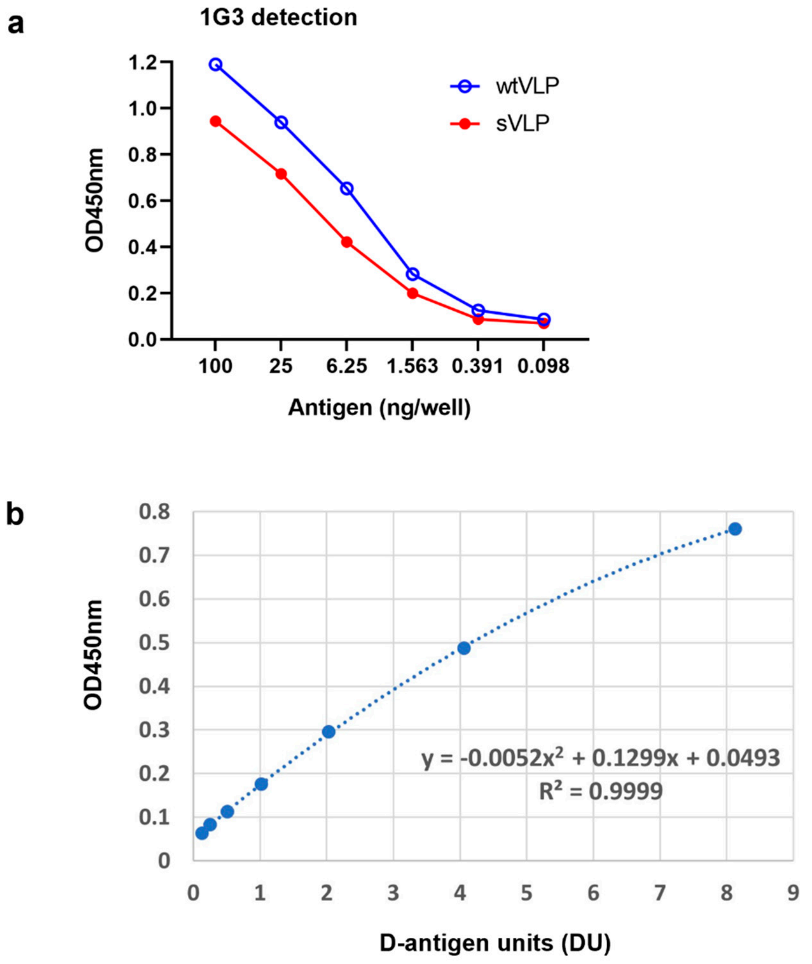

Supplementary Figure S1. D antigen quantitation by sandwich ELISA. (**a**) Mab 1G3 recognized both PV2 wtVLP and sVLP in ELISA. Representative data from three independent experiments were shown. (**b**) Standard curve generated by sandwich ELISA with the international IPV standard (NIBSC code: 12/104) as reference. Representative data from three independent experiments were shown.

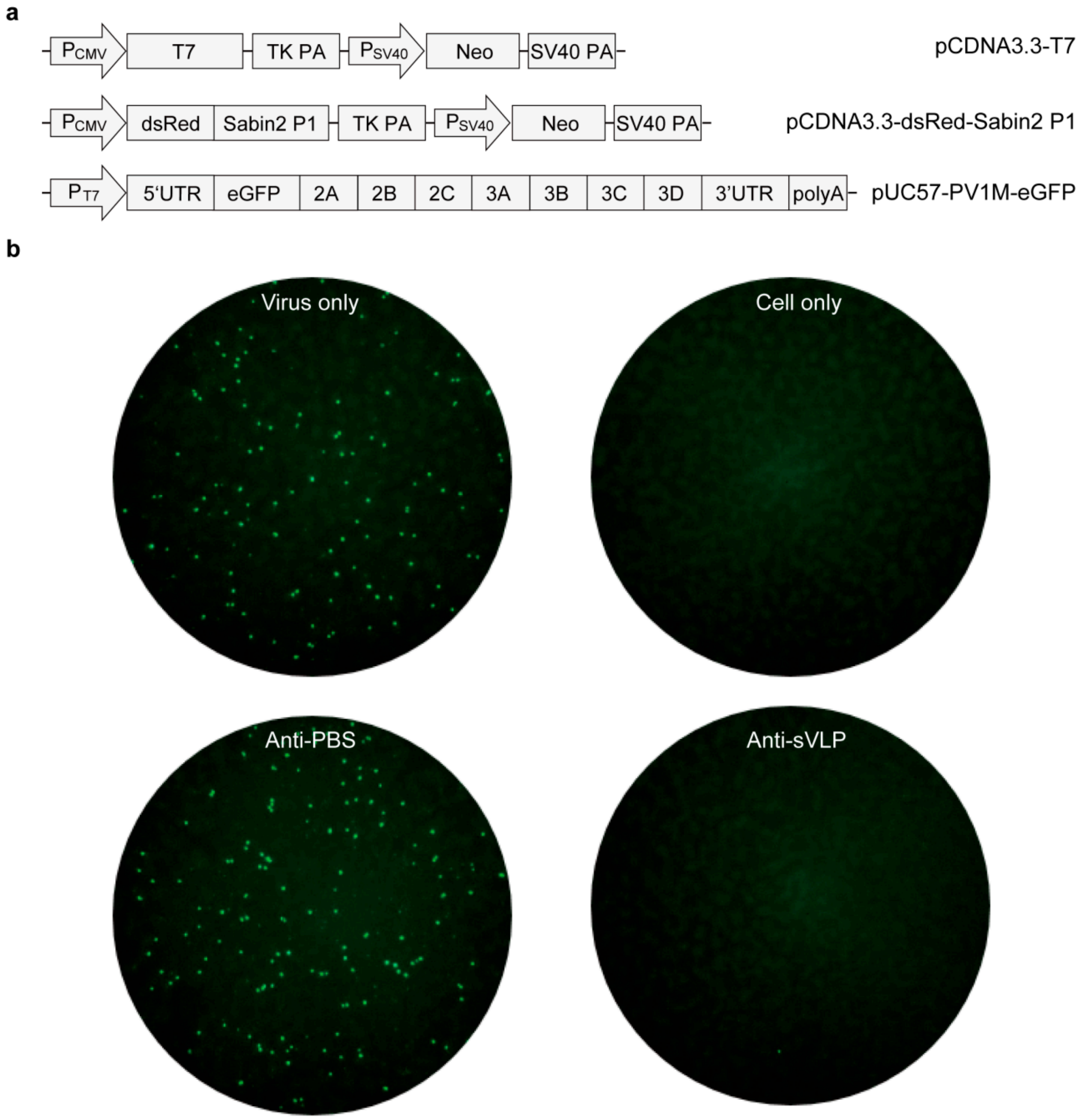

Supplementary Figure S2. PV2 pseudovirus neutralization assay. (**a**) Diagrams of the three plasmids used to generate the GFP-expressing PV2 pseudovirus. (**b**) Representative fluorescent images from neutralization experiments. Target cells were inoculated with PV2 pseudovirus ("Virus only") or with pseudovirus/antiserum mixtures ("Anti-sVLP" or "Anti-PBS"), incubated at 37°C for 20 hours, and then examined and imaged for GFP fluorescent spots using the ImmunoSpotS6 plate reader. Uninfected cells ("Cell only") were also examined to show background fluorescence.

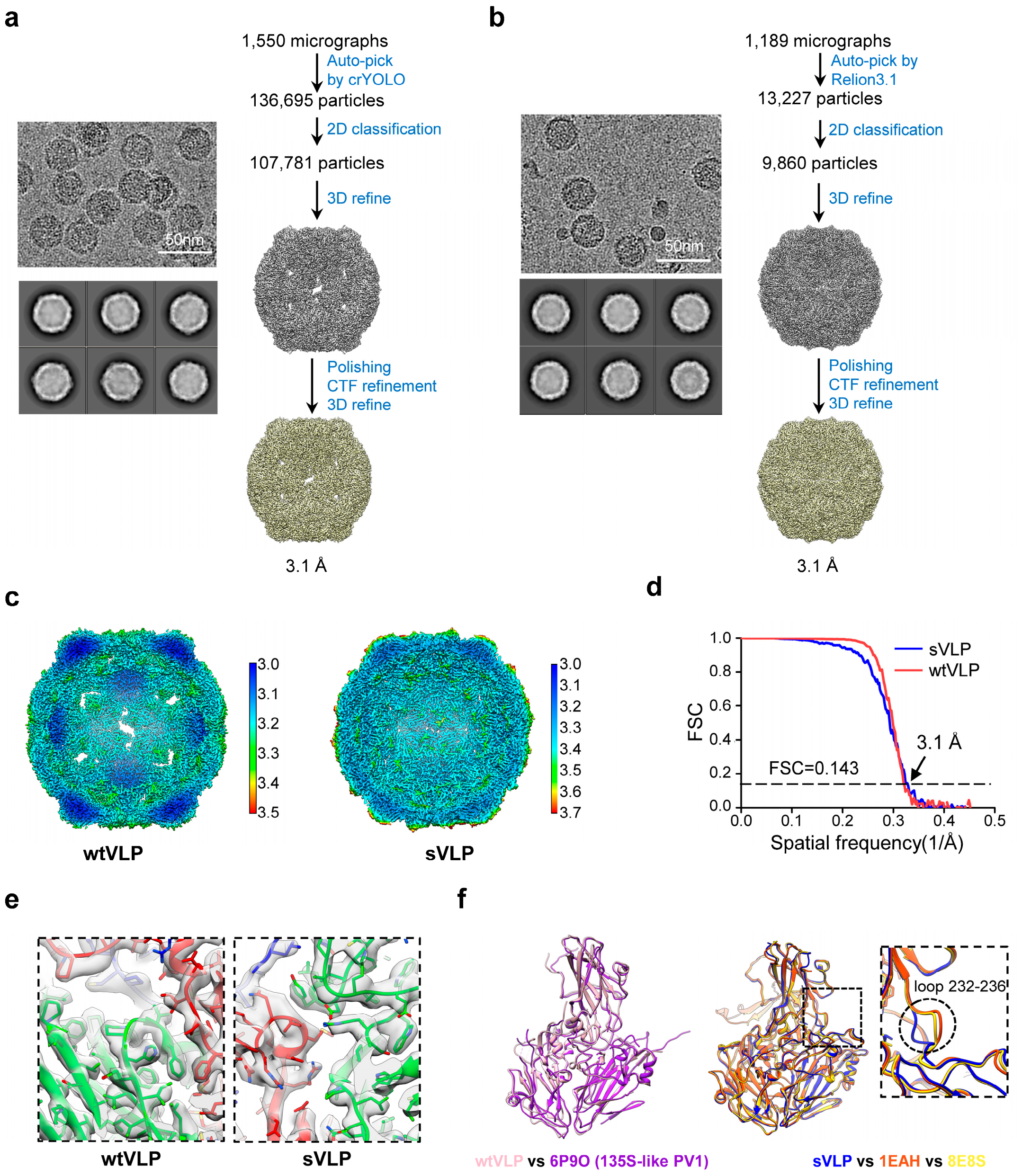

Supplementary Figure S3. Cryo-EM analysis of the VLPs.

(**a-b**) Data processing workflow for the wtVLP (**a**) and sVLP (**b**). A representative original micrograph and the reference-free 2D class averages are also presented. (**c**) Local resolution evaluations for the wtVLP and sVLP. (**d**) Resolution estimation of VLP maps according to the gold-standard FSD criterion of 0.143. (**e**) The high-resolution structure features for wtVLP and sVLP. (**f**). Structural comparison of wtVLP (in pale red) with PV1 expanded 135S particle structure (PDB: 6P9O, in purple) and sVLP (in blue) with mature PV2 (PDB: 1EAH, in orange red and PDB: 8E8S, in gold).

Supplementary Table S1. Cryo-EM data collection and refinement statistics for wtVLP and sVLP

	wtVLP	sVLP
**Data collection**		
EM equipment	Titan Krios	
Voltage (kV)	300	
Detector	Gatan K3 camera	
Pixel size (Å)	1.093	
Electron dose ($e^{-}/Å^{2}$)	50.2	
Exposure time (s)	3	
Frames	30	
Defocus range (μm)	-0.8 to -2.5	
**Reconstruction**		
Softwares	Relion 3.1	
Final particles	107,781	9,860
Symmetry	I1	I1
FSC threshold	0.143	
Final overall resolution (Å)	3.1	3.1
Resolution Range (Å)	3.0-3.3	3.0-3.6
Map sharpening B factor ($Å^{2}$)	-117.7	-61.7
**Atomic modeling**		
Softwares	Rosetta & Phenix & Coot	
Initial model ID	6P9O	8ayz
Bfactor range ($Å^{2}$)	34-83	30-70
Number of non-H atoms residues and ligands	4727, 0	5671, 1
Rms deviations		
Bond length (Å)	0.002	0.002
Bond Angle (°)	0.510	0.50
Ramachandran plot (%)		
Favored	96.06	97.48
Allowed	3.25	2.52
Outliers	0.68	0.00
Molprobity score	1.32	1.19
Clash score	2.68	2.95

Supplementary Table S2. Calculated interface area for the wtVLP and sVLP.

interface	interface area wtVLP → sVLP (Å^2)
Interface between protomers but within pentamer	
VP1/VP1	828.6→ 1009.6
VP1/VP3	181.7→ 200.3
VP2/VP3	351.9→ 578.3
VP3/VP3	443.3→ 788.7
Interface between pentamer	
VP2/VP2	0→ 22.7
VP2/VP3	786.4→ 1242.0
VP3/VP3	28.8→ 70.7
